# COVID-19 vaccine acceptance and its determinants in the Bono Region of Ghana

**DOI:** 10.4314/gmj.v56i4.2

**Published:** 2022-12

**Authors:** Bright T Forkuo, Joseph Osarfo, Gifty D Ampofo

**Affiliations:** 1 Department of Community Medicine, School of Medicine, University of Health and Allied Sciences, Ho, Ghana

**Keywords:** COVID-19, vaccine acceptance, determinants, Ghana

## Abstract

**Objective:**

The study assessed willingness to accept the COVID-19 vaccine among out-patient department (OPD) attendants in the Bono Region in Ghana.

**Design:**

This was an analytical cross-sectional study

**Setting:**

The study was conducted at the Wenchi Methodist Hospital (WMH) OPD, Bono Region, Ghana. The region had not yet been earmarked for vaccination at the time of the study.

**Participants:**

Three hundred and twenty-five (325) participants aged ≥18 years, accessing care at the OPD of WMH and willing to give informed consent, were interviewed.

**Main outcome measures:**

The proportion of participants willing to accept the COVID-19 vaccine and its determinants.

**Results:**

Of 325 participants interviewed, 32 (9.8%) had been vaccinated already. 82.6% (242/293) indicated COVID-19 vaccine acceptance among the unvaccinated**.** The major reason for vaccine acceptance was “it could protect against COVID-19” (96.7%, 234/242). “Fear of vaccine side effects and “perception of not being susceptible to COVID-19” were among the reasons for vaccine refusal. Perceived susceptibility to COVID-19 (AOR 4.09, 95% CI 1.79, 9.34), knowledge of COVID-19 and COVID-19 vaccine (AOR 3.62, 95% CI 1.14, 11.46) and willingness to pay for the vaccine (AOR 5.20, 95% CI 2.49, 10.43) were associated with vaccine acceptance.

**Conclusions:**

Adequate knowledge of COVID-19 and the vaccine may drive vaccine acceptance in the study area and possibly other areas in Ghana. Campaign messages aimed at increasing COVID-19 vaccine coverage must emphasise its safety, likely side effects and management in order to help rid the population of misconceptions.

**Funding:**

None indicated

## Introduction

Coronavirus Disease 2019 (COVID-19) is caused by severe acute respiratory syndrome coronavirus-2 (SARS-CoV-2); a new strain of virus belonging to the coronavirus family. It first started in Wuhan, China in December 2019 and later declared a pandemic by the World Health Organization (WHO) in March 2020. By late October 2021, over 246 million cases and close to five million deaths had been reported globally.^1^

Although other measures, including frequent hand washing using soap under running water, social distancing and wearing of face masks, are advocated, vaccines remain the most effective preventive tool for reducing disease transmission, morbidity and mortality^2^. Still, their acceptance has been shown to be variable.^3^–^6^ Reasons for low acceptance rates have included safety concerns, doubts about efficacy, anxiety over perceived harmful effects on fertility and politically-driven media content, among others.^7^–^9^ Male gender, older age and higher education levels have been reported as favourable factors for vaccine acceptance.^9^,^10^ Low COVID-19 vaccine acceptance will likely hinder efforts at achieving herd immunity and leave many at risk of clinically manifest and severe disease. This will likely prolong the pandemic and its economic burden.^11^

Ghana reported its first two cases of COVID-19 on 12th March, 2020. By the second week of June 2021, 1239 active cases and 793 deaths with close to 93,000 recoveries had been recorded.^12^ The administration of COVID-19 vaccines in Ghana began in March 2021 and followed a phased plan that initially targeted health workers and the elderly population (≥60 years of age) in parts of the Greater Accra Region, parts of the Central Region bordering Greater Accra and the Ashanti Region as these areas had the highest burden of cases. By the third week in May 2021, nearly 97% of a targeted 361,374 people in the areas mentioned had been vaccinated in the second round of the first phase in the vaccine roll-out.^13^

The literature review showed only two published studies that have reported COVID-19 vaccine acceptance in Ghana in the general population, excluding health workers.^10^,^14^ In the first study,^14^ 54.1% of respondents were willing to take the vaccine, while 51% of urban respondents over 15 years of age were willing to accept the vaccine in the second study.^10^ Both studies were conducted prior to vaccine deployment in the country. They thus did not reflect potential changes in vaccine acceptance rates that may occur in the context of actual deployment. It is possible that attitudes may change when unvaccinated people observe that those vaccinated did not suffer certain ‘expected’ side effects as they may have erroneously assimilated. Furthermore, both studies utilized web-based data collection tools, an approach that lends to selection bias and limits generalizability despite its practicality.

With at least 3 months of vaccine administration implemented by mid-June 2021, a re-assessment of COVID-19 vaccine acceptance was deemed important to inform on potential changes in vaccine acceptance and their implications for educational messages against vaccine hesitancy. This was considered against the background that not all parts of the country had access to the vaccines concurrently. A cross-sectional study to assess COVID-19 vaccine acceptance and influencing factors was conducted among outpatient department (OPD) attendants at the Wenchi Methodist Hospital in the Bono Region of Ghana.

## Methods

### Study design, population and setting

This was an analytical cross-sectional study among OPD attendants accessing care at the Wenchi Methodist Hospital in the Bono Region. The Wenchi Methodist Hospital (WMH) is the district hospital for the Wenchi Municipality in the Bono Region of Ghana. It is the main referral point for surrounding health facilities in the area. The average monthly OPD attendance is about 7,500 with about 200-250 people accessing care at the OPD daily. Over 80,000 attendants were recorded in 2020 (Health Information Unit, WMH, 2021). By May 2021, the Bono region had recorded 1410 confirmed cases of COVID-19, 1369 recoveries, 39 deaths and only 2 active cases**.**^12^

### Sample size determination

The sample size was estimated using the Cochran formula N=Z^2^pq/d^2^, where Z is the reliability coefficient of 1.96 for a 95% confidence interval, p is the proportion of people indicating acceptance of the COVID-19 vaccine, q is (1-p), and d is precision. Assuming a vaccine acceptance rate of 54.1%^14^ and a precision of 5%, a sample size of 382 was obtained.

### Data collection

Study participants were selected using a systematic random sampling approach. Assuming an average daily OPD attendance of 200 (see the study site description) and aiming to recruit 20 participants daily, a sampling interval of 10 was used. Data was collected on weekdays from 21st June, 2021 to 16th July, 2021 through face-to-face interviews using pretested structured questionnaires administered in English and Twi language where the participant could neither read nor write in English. Participants were included if they were at least 18 years old and willing to give informed consent. Clients who looked ill were excluded and not approached. On every interview day, every tenth client reporting at the nurses' station to have their blood pressure, temperature and pulse rate checked was approached and introduced to the study and its objectives. Where a client refused participation, the next client was approached and counting continued from that client. Interviews were conducted before or after clinical consultation from 9 am to 3 pm each day.

The questionnaire (see Supplementary file) was developed by the investigators based on a review of the literature. The questionnaire captured information on socio-demographic characteristics, including age, sex, level of education, marital status, employment status, religion, residence and income level. Level of knowledge of COVID-19 and COVID-19 vaccine, perceived reality of the existence of COVID-19, perceived susceptibility to COVID-19 and willingness to pay for the vaccine if there is a need to do so were also explored.

The dependent variable, acceptance of the COVID-19 vaccine if it was made available to the client, was characterised by a binary response of YES or NO. The level of knowledge of COVID-19 and the COVID-19 vaccine was assessed based on 11 questions. Participants scored 1 for a correct answer and 0 for a wrong answer or an ‘I do not know’ response. For those questions that allowed multiple responses, a score of 1 was still assigned once the selected responses included the correct answer. In contrast, 0 was assigned if the selected responses excluded the correct answer. A score of ≥ 6/11 was graded as ‘adequate knowledge’ while a score of ≥ 6/11 was ‘inadequate knowledge. The decision to use 6 as a cut-off was arbitrary and was informed by a need for the Ghanaian population to have a reasonably adequate knowledge of COVID-19 and the COVID-19 vaccine. No sampling weights were applied to any of the knowledge assessment questions as they were all deemed equally relevant. Responses for perceived susceptibility to COVID-19, perceived reality of the existence of COVID–19 and willingness to pay for vaccines were assessed as binary variables YES or NO.

### Data management and analysis

Data was double-entered in Microsoft Excel 2016 (Microsoft Corporation, Redmond, USA), cleaned and exported into STATA 13 (College Station, TX, USA) for analysis. Descriptive statistics were reported as frequencies and percentages. Association between dependent and independent variables was assessed using Chi-square and Fisher's exact tests as appropriate and logistic regression analysis. Independent variables with a statistically significant association in the bivariate analysis were entered into a multivariate logistic regression model. Odds ratios were reported with 95% confidence intervals, and statistical significance pegged at p<0.05.

### Ethical consideration

Ethical approval for the study was granted by the Research Ethics Committee (REC) of the University of Health and Allied Sciences, Ho, Ghana (reference number UHAS-REC A.12 [86] 2020-2021). Permission was also obtained from the management of WMH prior to the commencement of the study. Written informed consent was obtained from the participants. They were assured that participation was voluntary and that they could withdraw from the study even after agreeing to participate with no punitive measure such as denial of health care. Data were anonymized using study codes to ensure confidentiality. Wearing of face masks and social distancing were ensured for the interviewer and respondents during the questionnaire administration.

## Results

Three hundred and twenty-five (325) participants out of the estimated sample size of 382 could be recruited due to time constraints resulting in a recruitment rate of 85.1%. Of the recruited participants, 9.8% (32) had received the COVID-19 vaccine at the time of administering the questionnaire, even though the Bono Region was not designated for vaccine deployment at that time. These were excluded from further analysis in the description of the socio-demographic characteristics, assessment of vaccine acceptance and its determinants.

### Socio-demographic characteristics of study participants

Table 1 shows the socio-demographic characteristics of the study participants. The mean age (SD) was 45.6 years (17.4). More than half were females (53.2%, 156/293). Three-fifths (60.4%, 177/293) of the participants were married, and the dominant religion was Christianity (80.5%, 236/293). A little over a fifth (22.5%, 66/293) either had no formal education or had only a basic level of education. Most respondents (68.6%, 201/293) resided in relatively urbanised areas.

**Table 1:** Socio-demographic characteristics, knowledge and perceptions of COVID-19 and willingness to pay for COVID-19 vaccine of study participants

Variable	Acceptance of Covid		p-value
	YES (N=242)	NO (N=51)	
	n (%)	n (%)	
**Age**			**0.002**
<35 years	69 (28.5)	26 (51.0)	
≥35 years	173 (71.5)	25 (49.0)	
**Level of Education**			0.254
None/basic	57 (23.5)	9 (17.6)	
High School	121 (50.0)	32 (62.8)	
Tertiary	64 (26.5)	10 (19.6)	
**Employment Status**			0.621
Unemployed	70 (28.9)	13 (25.5)	
Employed	172 (71.1)	38 (74.5)	
**Sex**			0.057
Female	135 (55.8)	21 (41.2)	
Male	107 (44.2)	30 (58.8)	
**Marital Status**			**0.022** a
Divorced/widowed	38 (15.7)	3 (5.9)	
Single	55 (22.7)	20 (39.2)	
Married	149 (61.6)	28 (54.9)	
**Religion**			0.505^a^
Christian	197 (81.4)	39 (76.5)	
Moslem	42 (17.4)	11 (21.5)	
*Others	3 (1.2)	1 (2.0)	
**Residence**			0.736
Rural	77 (31.8)	15 (29.4)	
Urban	165 (68.2)	36 (70.6)	
**Income (GHS)**			0.420
<1000 / month	86 (45.5)	15 (38.5)	
≥1000 / month	103 (54.5)	24 (61.5)	
**Knowledge of COVID-19 and COVID-19 vaccine**			**< 0.001**
Inadequate knowledge	9 (3.7)	10 (19.6)	
Adequate knowledge	233 (96.3)	41 (80.4)	
**Perceived susceptibility to COVID-19**			**< 0.001**
Not susceptible	21 (8.7)	19 (37.3)	
Susceptible	221 (91.3)	32 (62.7)	
**Perceived reality of COVID-19**			**0.002**
COVID-19 is not real	36 (14.9)	17 (33.3)	
COVID-19 is real	206 (85.1)	34 (66.7)	
**Willingness to pay for COVID-19 vaccine**			**< 0.001**
No	37 (15.3)	31 (60.8)	
Yes	205 (84.7)	20 (39.2)	

a p-value from Fisher's exact test

* refers to traditional and the oriental religions

### Willingness to accept the COVID-19 vaccine

At least four-fifths (82.6%, 242/293) indicated acceptance of the vaccine if it is made available to them (see Table 1). In response to the main reasons for their acceptance of the vaccine ‘protection against COVID-19’ (96.7%, 234/242), ‘fear of COVID-19’ (1.2%, 3/242) and because ‘it was compulsory to get vaccinated’ (2.1%, 5/242) were given.

In order to explore the majority of potential barriers to acceptance, participants were allowed to give multiple reasons why they would not accept the vaccine. These included ‘fear of side effects (70.6%, 36/51), ‘fear that the vaccines were made by the Western world to infect them with COVID-19’ (56.9%, 29/51), ‘not susceptible to getting COVID-19’ (35.3%, 18/51), ‘because COVID-19 is not real’ (25.5%, 13/51), ‘vaccine may be expensive’ (2.0%, 1/51), ‘fear of future effects’ (3.9%, 2/51) and ‘no reason at all’ (2.0%, 1/51).

### Factors associated with COVID-19 vaccine acceptance

Age (p=0.002) and marital status (p=0.022) were the only socio-demographic factors that were significantly associated with covid-19 vaccine acceptance in the chi-square analysis, although the sex of the participant almost assumed a statistically significant association (p=0.057). Level of knowledge of COVID-19 and COVID-19 vaccines (p<0.001), perception of susceptibility (p<0.001), perception of the reality of COVID-19 (p=0.002) and willingness to pay for the vaccine (p<0.001) were all significantly associated with willingness to accept the COVID-19 vaccine (see Table 1).

In the bivariate logistic regression analysis (see Table 2), participants aged at least 35 years were nearly three times more likely to indicate vaccine acceptance compared to those less than 35 years (OR 2.61, 95%CI 1.41, 4.83). Single participants were 78% less likely to accept the vaccine if it was made available (OR 0.22, 95% CI 0.06, 0.78) compared to the divorced/widowed. Participants who were married also had 58% fewer odds of accepting the vaccine (OR 0.42, 95% CI 0.12, 1.46), but this was not statistically significant.

**Table 2:** Logistic regression analysis output for an association between COVID-19 vaccine acceptance and assessed independent variables

Variable	*COR (95% CI)	p- value	**AOR (95% CI)	p-value
**Age (years)**				
<35	1		1	
≥35	2.61 (1.41, 4.83)	0.002	1.57 (0.75, 3.26)	0.229
					
**Marital Status**				
Divorced/widowed	1		1	
single	0.22 (0.06, 0.78)	0.020	0.91 (0.51, 1.58)	0.731
married	0.42 (0.12, 1.46)	0.171		
					
**Perceived reality of COVID-19**				
Covid is not real	1		1	
Covid is real	2.86 (1.45, 5.66)	0.002	1.45 (0.63, 3.32)	0.378
					
**Perceived susceptibility to COVID-19**				
Not susceptible	1		1	
Susceptible	6.25 (3.03, 12.87)	<0.001	4.09 (1.79, 9.34)	0.001
					
**Knowledge of COVID-19 and COVID-19 vaccine**				
Inadequate knowledge	1		1	
Adequate knowledge	6.31 (2.42, 16.49)	<0.001	3.62 (1.14, 11.46)	0.029
					
**Willingness to pay**				
No	1		1	
Yes	8.59 (4.43, 16.65)	<0.001	5.20 (2.49, 10.83)	<0.001

*COR=Crude Odds Ratio. **AOR= Adjusted Odds Ratio: each variable was adjusted for the rest of the variables shown in the table

Those who perceived they were susceptible to COVID-19 (OR 6.25, 95% CI 3.03, 12.87) and those with adequate knowledge of the disease and the vaccine (OR 6.31, 95% CI 2.42, 16.49) had at least six times the odds of accepting the vaccine compared to those who thought they were not susceptible to the disease and those with inadequate knowledge of the disease/vaccine respectively. Participants willing to pay for the vaccine if need be were about nine times as likely to accept the vaccine compared to those unwilling to pay for it (OR 8.59, 95% CI 4.43, 16.65).

In the multivariate logistic regression analysis, only participants' perceived susceptibility to COVID-19 (AOR 4.09, 95% CI 1.79, 9.34), knowledge of COVID-19 and COVID-19 vaccine (AOR 3.62, 955 CI 1.14, 11.46) and willingness to pay for the vaccine if need be (AOR 5.20, 95% CI 2.49, 10.43) remained significantly associated with vaccine acceptance (Table 2)

## Discussion

A cross-sectional study was conducted to assess COVID-19 vaccine acceptance and factors influencing it in a non-health worker population in an area of Ghana that was yet to access the vaccine in spite of vaccine roll-out in other parts of the country. A vaccine acceptance rate of 82.6% was observed in the study, and participants' own perceived risk of the disease, knowledge of COVID-19/COVID-19 vaccine and willingness to pay for the vaccine if need be were the factors driving acceptance.

The present study's 82.6% acceptance rate was higher than the 39.3% reported among health workers^15^ and the 54.1% and 51.0 % reported in two studies in the general population prior to COVID-19 vaccine deployment in Ghana.^14^,^10^

The finding was also higher than the pooled acceptance rate of 49% reported in a systematic review of studies conducted in Africa.^16^

The Bono Region was listed among the bottom five regions in Ghana likely to accept the COVID-19 vaccine with an acceptance rate of about 47% in a study^10^, and this was far less than that reported in the current study. The variation may lie in the different study methods used. The study sample used in the previous study was restricted to literates with access to the internet^10^. It is also possible that the population in the Bono Region have realised from other regions with vaccine access at the time of data collection that taking the vaccine is not harmful. The high vaccine acceptance observed in the present study may also be attributed to the survey conducted among people attending a health facility. Invariably, their positive health-seeking behaviour may have influenced their acceptance and hence a higher rate.

Compared to the Ghanaian studies,^10^,^14^ the high acceptance rate in the present study is also probably attributable to the public education on the importance of vaccination in this pandemic and disabusing their minds of some conspiracy theories that surrounded the vaccines initially. Similar high COVID-19 vaccine acceptance rates have been reported in Asia, Europe, and the Caribbean/Latin America, but these were mostly recorded before the availability of COVID-19 vaccines in those countries.^3^,^17^–^19^

The commonest reason underlying their willingness to accept the vaccine was the belief that it could protect them against the disease. It is likely that participants perceived COVID-19 as a threat and thus are more likely to accept the vaccine aimed at mitigating the effects of that threat.^8^ The reasons for vaccine refusal compare favourably with those reported earlier^20^,^21^ and appear global. The notion that the COVID-19 vaccine is intentionally meant to bring harm to recipients, including infection with the virus, was well grounded among African countries in a study of COVID-19 vaccine acceptance in low- and middle-income countries.^21^

In the present study, participants were categorised as having adequate or inadequate knowledge of COVID-19 and COVID-19 vaccines based on an arbitrary cut-off mark of 6/11, informed by the desired need for Ghanaians to have reasonably sufficient knowledge of the subject matter. In spite of its utility, this approach may not be entirely scientific and could pose challenges for comparison to other study findings. However, variations of this approach, including the use of cut-off marks above the median or mean score to signify adequate or good knowledge of the COVID-19 vaccine, have been observed in previous studies.^22^,^23^

Knowledge of the COVID-19 vaccine, which appears intertwined with participants' perception of their susceptibility, reality of COVID-19 and willingness to pay for the vaccine if need be, was found to drive vaccine acceptance in the current study. Knowledge of the disease almost quadrupled the odds of vaccine acceptance and is similar to findings reported in a survey among low and middle-income countries.^21^ Similarly, the findings relating to participants' perception of their own risk or susceptibility to the disease is aligned with other study findings.^14^ Close to 77% of respondents in the current study were willing to pay to get themselves vaccinated against COVID-19, similar to a study where 83% of respondents in a study in Chile expressed willingness to pay^24^ but contrary to a Nigerian study where only 18.4% of the respondents were willing to pay for the vaccine.^25^ Willingness to pay for the vaccine is underpinned by a good knowledge of the disease and an understanding that it can be prevented.

Age ≥35 years was positively associated with vaccine acceptance in the bivariate analysis, but this was lost in the multiple regression model. The other variables in the model likely confounded the initial association. The relationship between age and COVID-19 vaccine acceptance is variable as some studies have reported higher odds of acceptance with younger age^14^,^21^ with the opposite being the case in others.^20^ Marriage did not appear to be relevant to vaccine acceptance in the current study. However, another study in Ghana reported that married participants were less likely to accept the vaccine than single participants.^14^ The present study's finding, in the bivariate regression, that single participants were less likely to accept the vaccine may have been a result of confounding by age and other variables included in the multiple regression analysis.

Sex was not associated with vaccine acceptance in this study, but other studies reported that females had reduced odds of vaccine acceptance.^10^,^14^,^21^ Similarly, income and level of education did not impact on vaccine acceptance, and these compare favourably with findings reported in local surveys.^10^,^14^ One may presume that knowledge of COVID-19 and its varied dimensions expressed in the perception of susceptibility and reality of COVID-19 are the key drivers of COVID-19 vaccine acceptance in this period of vaccine roll-out and may explain why sex, income, level of education, or residence, were not associated with the outcome.

The study has a number of limitations. The inability to recruit the estimated sample size and excluding those vaccinated from analysis affected the sample size adversely and likely accounts for the reduced precision exhibited in the wide confidence intervals around some odds ratios.

In this context, the association between the variables ‘knowledge of COVID-19/COVID-19 vaccine’, ‘perceived susceptibility’ and ‘willingness to pay’ and the outcome of COVID-19 vaccine acceptance may need to be interpreted with caution. Secondly, though the study was more open to illiterates and people with limited formal education, recruiting hospital attendants as participants still introduces selection bias and limits generalizability. Data collection within communities for better sample representation would have increased study rigour. Nevertheless, the findings reported are relevant and highlight the need to continue reinforcing COVID-19 risk communication for citizens to adopt the appropriate preventive measures including the vaccine.

## Conclusion

COVID-19 risk perception, underpinned largely by knowledge of the disease and the vaccine, may be the driving factor for vaccine acceptance when vaccine roll-out begins in the study area. Campaign messages must focus on the safety and efficacy of the vaccines as these appear to be the main reasons for refusal. Further studies with community-based data collection methods will give more precise vaccine acceptance estimates.
